# Aortic valve calcification is subject to aortic stenosis severity and the underlying flow pattern

**DOI:** 10.1007/s00380-020-01688-9

**Published:** 2020-09-07

**Authors:** Verena Veulemans, Kerstin Piayda, Oliver Maier, Georg Bosbach, Amin Polzin, Katharina Hellhammer, Shazia Afzal, Kathrin Klein, Lisa Dannenberg, Saif Zako, Christian Jung, Ralf Westenfeld, Malte Kelm, Tobias Zeus

**Affiliations:** 1 Division of Cardiology, Pulmonology and Vascular Medicine, Heinrich Heine University, Moorenstr. 5, 40225 Düsseldorf, Germany; 2 CARID (Cardiovascular Research Institute Düsseldorf), Heinrich Heine University, Moorenstr. 5, 40225 Düsseldorf, Germany

**Keywords:** TAVR, TAVI, Aortic valve calcification, AS entities

## Abstract

Sex- and flow-related aortic valve calcification (AVC) studies are still limited in number, and data on the exact calcium quantity and distribution are scarce. Therefore, we aimed to (1) re-define the best threshold of AVC load to distinguish severe from moderate aortic stenosis (AS) in common AS entities and to (2) evaluate differences in the aortic annulus and left ventricular outflow tract (LVOT) calcium load. Nine hundred and thirty-eight patients with contrast-enhanced cardiac MSCT and moderate-to-severe aortic stenosis (AS) were retrospectively enrolled. Patients with severe AS ≤ 1.0 cm^2^ (*n* = 841) were further separated into three AS entities: high gradient (HGAS, *n* = 370, 44.0%), paradoxical low gradient (pLGAS, *n* = 333, 39.6%), and classical low gradient (LGAS, *n* = 138, 16.4%). AVC, leaflet, and LVOT calcification were quantified. Aortic valve calcification scores were highest in severe HGAS, and lower in severe pLGAS and classical LGAS. In all severity and AS entities, the non-coronary cusp (NCC) was the most calcified one. LVOT calcification was consistently comparable between gender and AS entities. Accuracy of logistic regression was the highest in HGAS (male vs. female: AVC > 2156 Agatston units (AU), c-index 0.76; vs. AVC > 1292 AU, c-index 0.85; or AVC density > 406 AU/cm^2^, c-index 0.82; vs. > 259 AU/cm^2^, c-index 0.86; each *p* < 0.0001*) to diagnose severe AS. AVC could only be used in men to differentiate between severe LGAS and moderate AS. Data from this retrospective analysis indicate that the NCC is subject to pre-dominant degeneration throughout gender, AS severity, and several AS entities. AVC was consistently comparable in severe pLGAS and classical LGAS, but only AVC in severe LGAS could sufficiently distinguish from moderate AS in men. LVOT calcification failed to be a reliable indicator of accelerating AS.

**Electronic supplementary material:**

The online version of this article (10.1007/s00380-020-01688-9) contains supplementary material, which is available to authorized users.

## Introduction

Aortic valve stenosis (AS) is the most common valvular heart disease in western countries with increasing prevalence [1]. Previous studies have demonstrated that the extent of aortic valve calcification (AVC), measured by multi-slice computed tomography (MSCT), correlates well with the hemodynamic severity of AS [2, 3]. If compared to their male counterparts, woman tend to reach a higher degree of stenosis for the same amount of AVC burden, even after adjusting for body surface area and smaller aortic annulus size [4, 5]. Furthermore, different AS entities are associated with discordant gradients from the aortic valve area (AVA), based on multiple valvular and non-valvular factors not depending on flow [4]. AVC quantification by MSCT helps to identify patients with severe AS: AVC > 1274 AU in women and 2065 AU in men or with AVC density (indexed to annulus cross-sectional area) > 292 AU/cm^2^ in women and > 476 AU/cm^2^ in men are set to be the cut-off to distinguish between moderate and severe AS [4]. Those findings entered current guideline recommendations on the management of patients with valvular heart disease to improve clinical decision-making in patients with inconsistent diagnostic findings.

However, the definition of AS entities is subject to continuous modifications, and the existing sex- and flow-related AVC studies are limited to the number of studies in this context, and detailed information about calcium distribution and severity in patients with altered flow patterns are still missing. Therefore, we aimed to (1) re-define the best threshold of AVC load to distinguish severe from moderate AS in several AS entities and to (2) evaluate differences in the calcium load of the aortic annulus and left-ventricular outflow tract (LVOT).

## Methods

### Study population

We retrospectively enrolled 938 consecutive patients with moderate-to-severe tricuspid AS, who underwent diagnostic work-up for transcatheter aortic valve replacement between 2011 and 2019 at our heart center. Patients with moderate-to-severe AS underwent MSCT if findings were heterogeneous and severe AS was clinically suspected. This especially applies to low-flow conditions and is in accordance with the current guideline recommendations for the treatment of patients with valvular heart disease. Those consider the calcium burden as a key decision-making factor if uncertainty in the grading of AS still exists after extensive work-up. AS severity was defined according to the existing guidelines [6]. Patients were further grouped into three AS entities, according to their flow pattern, as mentioned below. Patients with overt rheumatic valve disease, endocarditis, bicuspid morphology, or prior aortic valve replacement were excluded to account for comparability.

All patients provided written informed consent for the use of clinical, procedural, and follow-up data. The study procedures were in accordance with the Declaration of Helsinki and the institutional Ethics Committee of the Heinrich-Heine University approved the study protocol (4080). The study is registered at clinical trials (NCT01805739).

### Statistical analysis

The collected data included patient characteristics and imaging findings. Continuous data were described as mean with standard deviation, median or upper and lower 95% confidence interval, and interquartile ranges. Categorical variables were expressed by frequencies and percentages. Differences between men and women were analyzed with the use of the two-sided Student’s *t* test for continuous variables and the Fisher’s exact test for categorical variable. To compare continuous variables without normal distribution, we used the Mann–Whitney *U* test. The impact of sex was assessed by the interaction between sex and stenotic indexes in correlation, using transformed and untransformed AVC. One-way ANOVA with Tukey post hoc analysis was used to compare differences between more than two groups. Receiver-operating characteristic (ROC) analysis and the c-index (area under the curve, AUC) were used to identify the sensitivity and specificity of the AVC thresholds defined by AS severities and AS entities. The optimal cut-off values were defined by Youden’s index, the point at which the value of “sensitivity + specificity − 1” was maximal.

The data analysis was performed using the statistical software SPSS (version 22.0, SPSS Inc., Chicago, IL, USA) and GraphPad Prism (version 6.0, Graphpad Software, San Diego, CA, USA). All statistical tests were two-tailed, and a value of *p* < 0.05 was considered statistically significant.

### Imaging modalities

#### Transthoracic echocardiography (TTE)

Transthoracic echocardiography was performed according to current expert recommendations. Severe high-gradient AS (HGAS) was defined as normal left-ventricular function (LVF) > 50% with high gradients (mean gradient > 40 mmHg) and a calculated AVA below 1.0 cm^2^. Paradoxical severe low-gradient AS (pLGAS) was defined as preserved LVF > 50% combined with a mean gradient < 40 mmHg and a calculated AVA below 1.0 cm^2^. Classical severe low-gradient aortic stenosis (LGAS) was defined as reduced LVF < 50% combined with a mean gradient < 40 mmHg and a calculated AVA below 1.0 cm^2^. An AVA above 1.0–1.5 cm^2^ was defined as moderate AS. The true severity of AS was determined—especially in dis-concordant borderline AS—by multiple validating tools according to the current recommendations (transesophageal echocardiography, cardiac magnetic resonance tomography, dobutamine stress echo if indicated, and MSCT-derived calcification load) and the final decision was made in the interdisciplinary heart team.

#### 3D image analysis of MSCT

Cardiac CT was routinely performed as native and contrast-enhanced multi-slice CT. CT data were obtained using a 128-slice, single source CT scanner with temporal resolution of 150 ms and a collimation of 128 × 0.6 mm (“SOMATOM Definition AS+”, Siemens Healthcare, Forchheim, Germany) according to TAVR-related standardized recommendations for CT image acquisition [7]. Images were analyzed in the diastolic phase. MSCT data were transferred to a dedicated workstation for three-dimensional (3D) volume-rendered reconstruction (3mensio Structural Heart™, Pie Medical Imaging BV, Maastricht, The Netherlands). Dimensions were determined with the use of workstation tools. The total AVC and calcium amount of the upper LVOT are expressed as recalculated Agatston units (AU) adapted from the calcium volume and subsequently divided by the MSCT-derived annulus area to estimate calcium density (AU/cm^2^). Every area section was handled separately (LVOT, AVC, leaflets) concerning the calcium amount and according to current recommendations. A pre-specific threshold of at least 600 HU was set to account for the hyperdensity of the applied contrast medium as practicable approach according to current research data [8]. All MSCT-reconstructions and depending analyses were done by experienced level 3 readers. In general, upper and lower levels were defined according to the median and interquartile range.

## Results

### Baseline characteristics

443 male (47.2%) and 495 female (52.8%) patients were included. Male patients were younger (80.4 ± 5.8 years vs 82.3 ± 5.4 years; *p* < 0.0001) and presented more often with concomitant coronary (CAD: 80.6% vs 64.9%; *p* < 0.0001) and peripheral artery disease (PAD: 35.4% vs 23.0%; *p* < 0.0001). Previous percutaneous coronary intervention (PCI: 47.0% vs 31.3%; *p* < 0.0001), and coronary artery bypass grafting (CABG: 22.1% vs 4.5%; *p* < 0.0001) was more frequent in men than in woman. Left-ventricular ejection fraction was lower in male patients (LVEF: 52.0 ± 13.4% vs 58.2 ± 12.7%; *p* < 0.0001), accompanied by lower mean aortic valve gradients (dPmean: 35.7 ± 14.9 mmHg vs 38.6 ± 16.7 mmHg; *p* = 0.005). Further baseline characteristics are displayed in Supplemental material—Table 1.

### AVC thresholds according to AS severity

**Fig. 1:**
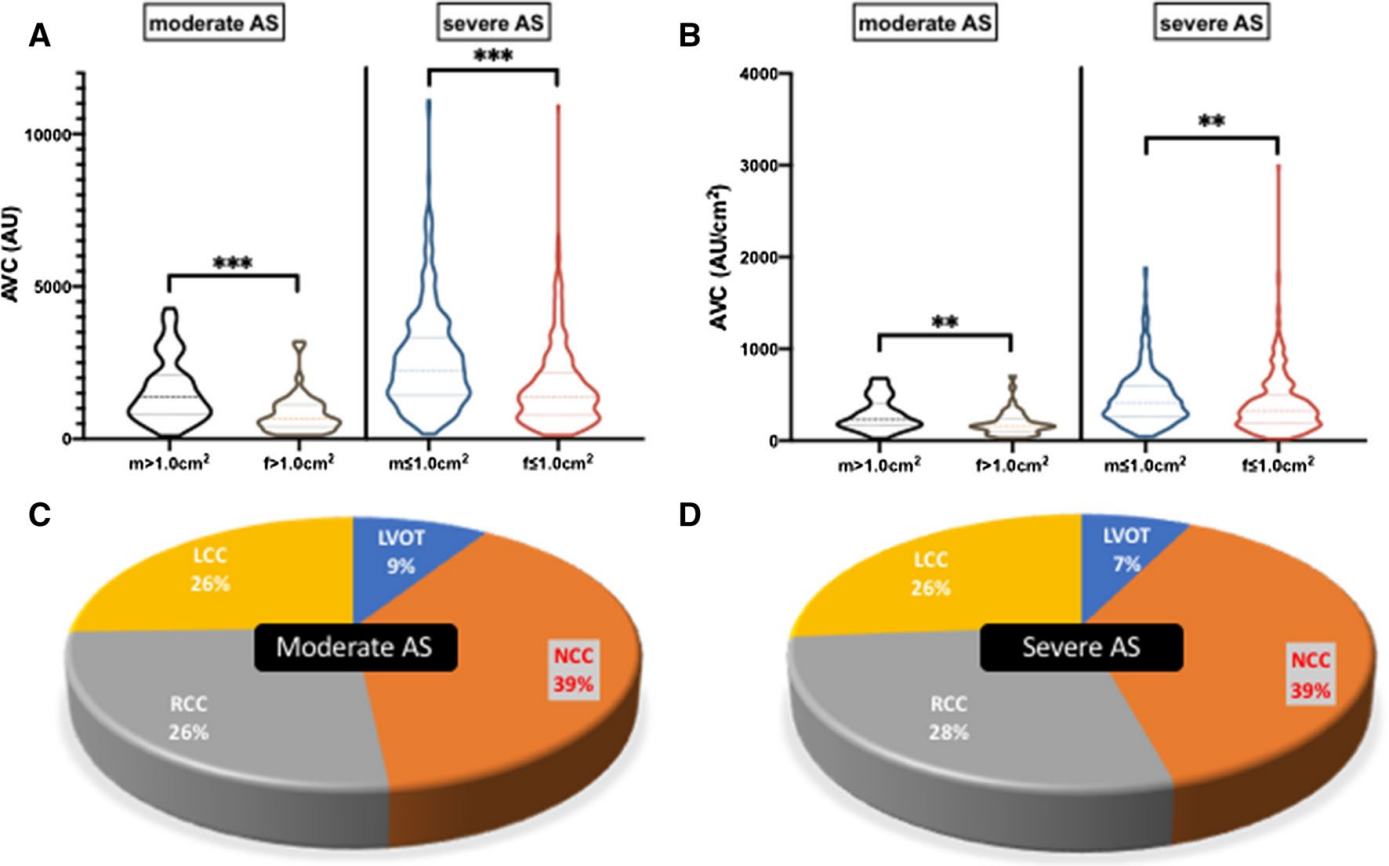
**a** Total AVC in AU calculated in moderate and severe AS in comparison of male and female patients. **b** Calcium density (AU/cm^2^) proportions in comparison of male and female patients in moderate and severe AS. **c** Distribution of calcium load in moderate AS and **d** severe AS

Nine hundred and thirty-eight patients were separated into moderate (AVA > 1 cm^2^, *n* = 97; 10.3%) and severe (AVA ≤ 1.0 cm^2^, *n* = 841; 89.7%) AS. AVC scores were higher in male as compared to female patients in terms of the total calcium aortic valve burden, which was separated into the distinctive leaflet calcium burden, after recalculating in density proportion. AVC thresholds almost doubled if moderate AS was compared to severe AS (male: AVA > 1 cm^2^/ ≤ 1.0 cm^2^: 1365 AU [827–2106] vs. 2245 AU [1418–3340]; *p* < 0.0001*; female: AVA > 1 cm^2^/ ≤ 1.0 cm^2^: 642 AU [407–1124] vs. 1388 AU [772–2187]; *p* < 0.0001*; Table 1, Fig. 1a, b). In all groups, the non-coronary cusp (NCC) was the most calcified one (Fig. 1c, d). LVOT calcification, in total and recalculated as density proportion in AU/cm^2^, was comparable throughout sex, but different in female patients concerning AS severity grading.

**Table 1:** Aortic stenosis quantification and associated calcification

Echocardiography and MSCT M = male; F = female	AVA > 1.0 cm^2^ (*n* = 97) M = 53; F = 44	*p* value	AVA ≤ 1.0 cm^2^ (*n* = 841) M = 390; F = 451	*p* value	*p*_AS-severity_
M AVA, cm^2^	1.2 ± 0.1	0.069	0.7 ± 0.2	< 0.0001*	< 0.0001*
F AVA, cm^2^	1.1 ± 0.1		0.7 ± 0.2		< 0.0001*
M dPmean, mmHg	25.1 ± 8.8	0.359	37.1 ± 15.0	0.007*	< 0.0001*
F dPmean, mmHg	23.4 ± 9.6		40.1 ± 16.6		< 0.0001*
M LVEF, %	49.5 ± 14.6	0.007*	52.2 ± 13.3	< 0.0001*	0.292
F LVEF, %	60.5 ± 14.5		58.0 ± 12.5		0.334
M AVC, AU	1365 [827–2106]	< 0.0001*	2245 [1418–3340]	< 0.0001*	< 0.0001*
F AVC, AU	642 [407–1124]		1388 [772–2187]		< 0.0001*
M AVC density, AU/cm^2^	228 [162–411]	0.002*	414 [265–602]	< 0.0001*	< 0.0001*
F AVC density, AU/cm^2^	157 [98–243]		322 [184–492]		< 0.0001*
M NCC, AU	482 [283–1025]	< 0.0001*	868 [502–1364]	< 0.0001*	0.001*
F NCC, AU	241 [89–433]		572 [268–1042]		< 0.0001*
M NCC density, AU/cm^2^	95 [54–173]	0.001*	158 [95–253]	0.001*	< 0.0001*
F NCC density, AU/cm^2^	49 [25–104]		132 [65–235]		< 0.0001*
M RCC, AU	361 [159–641]	< 0.0001*	641 [362–1085]	< 0.0001*	< 0.0001*
F RCC, AU	170 [48–360]		364 [200–652]		< 0.0001*
M RCC density, AU/cm^2^	65 [29–131]	0.019*	113 [65–195]	< 0.0001*	< 0.0001*
F RCC density, AU/cm^2^	45 [12–76]		83 [44–155]		< 0.0001*
M LCC, AU	330 [160–571]	0.002*	607 [329–1032]	< 0.0001*	< 0.0001*
F LCC, AU	193 [79–309]		326 [165–626]		0.0001*
M LCC density, AU/cm^2^	65 [36–100]	0.055	111 [61–178]	< 0.0001*	< 0.0001*
F LCC density, AU/cm^2^	46 [21–71]		79 [38–145]		< 0.0001*
M LVOT, AU	15 [0–157]	0.202	32 [0–176]	0.820	0.661
F LVOT, AU	4 [0–75]		34 [2–189]		0.027*
M LVOT density, AU/cm^2^	4 [0–24]	0.272	6 [0–30]	0.266	0.623
F LVOT density, AU/cm^2^	1 [0–19]		6 [0–42]		0.023*

Values are mean ± SD, median ± interquartile range or *n* (%)

*AVA* aortic valve area, *AVC* aortic valve calcification, *AU* Agatston units, *dPmean* mean transvalvular gradient, *LCC* left coronary cusp, *LVEF* left-ventricular ejection fraction, *LVOT* left -ventricular outflow tract, *NCC* non-coronary cusp, *RCC* right coronary cusp

* *p* < 0.05

C-statistics (Supplemental material—Table 2) depicted an AVC > 2020 AU in men (c-index 0.70, 95% CI 0.62–0.77; *p* < 0.0001*) and > 1137 AU in women (c-index 0.75, 95% CI 0.68–0.82; *p* < 0.0001*) or AVC density > 323 AU/cm^2^ in men (c-index 0.70, 95% CI 0.63–0.78; *p* < 0.0001*) and > 202 AU/cm^2^ in women (c-index 0.75, 95% CI 0.69–0.82; *p* < 0.0001*) as the best threshold for diagnosis of severe AS. NCC density proportion performed best in female patients to identify severe AS, and LCC density proportion in male, respectively. LVOT calcification failed to be a reliable indicator for severe AS.

### AVC thresholds according to AS entity

**Fig. 2:**
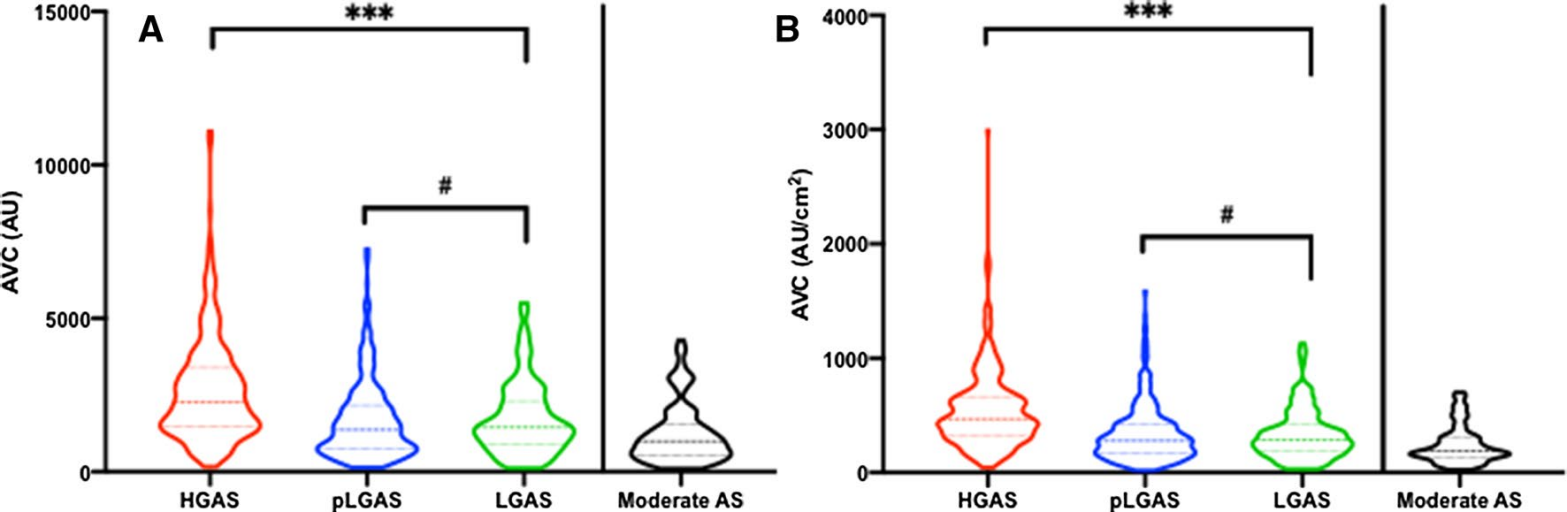
Overall calcium distribution in several AS entities compared to moderate AS. **a** Total AVC in AU calculated in severe HGAS, pLGAS, and LGAS compared to moderate AS. **b** Calcium density (AU/cm^2^) proportions in severe HGAS, pLGAS, and LGAS compared to moderate AS

Eight hundred and forty-one patients with severe AS were further separated into HGAS (*n* = 370; 44.0%), pLGAS (*n* = 333; 39.6%), and LGAS (*n* = 138; 16.4%). Related hemodynamic profiles to the underlying flow patterns are displayed in Table 2. AVC scores were higher in male than in female patients and highest in HGAS (male vs. female: 3076 AU [2211–3884] vs. 1785 AU [1237–2720], *p* < 0.0001*; 551 AU/cm^2^ [401–707] vs. 424 AU/cm^2^ [292–625], *p* = 0.001*). AVC thresholds were comparable in pLG and LGAS throughout gender, including the total AU and density proportions (Table 2, Fig. 2a, b). In all groups, the NCC was the most calcified cusp.

**Table 2:** Hemodynamics and AVC in severe AS and in dependency of AS entity

Echocardiography and MSCT M = male; F = female	HGAS (*n* = 370) M = 156; F = 214	*p* value	pLGAS (*n* = 333) M = 144; F = 189	*p* value	LGAS (*n* = 138) M = 90; F = 48	*p* value	pANOVA
M LVEF, %	55.2 ± 12.9^#^	0.002*	58.5 ± 8.3^#^	0.027*	35.9 ± 10.1	0.499	< 0.0001* n.s^#^
F LVEF, %	60.3 ± 11.4^#^		61.1 ± 8.5^#^		37.4 ± 10.3		< 0.0001* n.s^#^
M AVA, cm^2^	0.7 ± 0.2	< 0.0001*	0.8 ± 0.2^#^	0.130	0.8 ± 0.1^#^	0.108	< 0.0001* n.s^#^
F AVA, cm^2^	0.6 ± 0.2		0.8 ± 0.1^#^		0.7 ± 0.1^#^		< 0.0001* n.s^#^
M dPmean, mmHg	51.3 ± 11.7	0.045*	29.0 ± 7.0	0.369	25.7 ± 8.4	0.822	< 0.0001*
F dPmean, mmHg	53.8 ± 12.2		28.2 ± 7.5		25.4 ± 8.6		< 0.0001*
M AVC, AU	3076 [2211–3884]	< 0.0001*	1893 [1085–2715]^#^	< 0.0001*	1644 [1127–2639]^#^	< 0.0001*	< 0.0001* n.s^#^
F AVC, AU	1785 [1237–2720]		1014 [610–1666]^#^		1007 [521–1547]^#^		< 0.0001* n.s^#^
M AVC density, AU/cm^2^	551 [401–707]	0.001*	333 [216–481]^#^	< 0.0001*	322 [220–490]^#^	0.004*	< 0.0001* n.s^#^
F AVC density, AU/cm^2^	424 [292–625]		244 [144–389]^#^		230 [123–340]^#^		< 0.0001* n.s^#^
M NCC, AU	1138 [737–1789]	< 0.0001*	703 [424–1224]^#^	< 0.0001*	631 [407–1086]^#^	0.005*	< 0.0001* n.s^#^
F NCC, AU	751 [437–1228]		379 [169–731]^#^		416 [238–753]^#^		< 0.0001* n.s^#^
M NCC density, AU/cm^2^	206 [134–308]	0.045*	125 [82–213]^#^	< 0.0001*	124 [76–204]^#^	0.121	< 0.0001* n.s^#^
F NCC density, AU/cm^2^	175 [97–288]		92 [41–153]^#^		104 [62–169]^#^		< 0.0001* n.s^#^
M RCC, AU	970 [493–1389]	< 0.0001*	495 [246–844]^#^	< 0.0001*	538 [296–832]^#^	< 0.0001*	< 0.0001* n.s^#^
F RCC, AU	534 [276–800]		280 [151–519]^#^		232 [103–382]^#^		< 0.0001* n.s^#^
M RCC density, AU/cm^2^	167 [98–244]	< 0.0001*	93 [51–142]^#^	0.003*	97 [56–148]^#^	< 0.0001*	< 0.0001* n.s^#^
F RCC density, AU/cm^2^	126 [70–199]		68 [34–115]^#^		52 [27–78]^#^		< 0.0001* n.s^#^
M LCC, AU	822 [521–1172]	< 0.0001*	456 [264–7800]^#^	< 0.0001*	500 [261–723]^#^	< 0.0001*	< 0.0001* n.s^#^
F LCC, AU	452 [254–811]		244 [131–442]^#^		208 [97–428]^#^		0.0003* n.s^#^
M LCC density, AU/cm^2^	148 [97–210]	0.010*	83 [48–151]^#^	< 0.0001*	96 [51–131]^#^	0.001*	< 0.0001* n.s^#^
F LCC density, AU/cm^2^	108 [59–182]		55 [30–104]^#^		50 [24–99]^#^		< 0.0001* n.s^#^
M LVOT, AU	55 [0–260]^#^	0.761	33 [0–151]^#^	0.392	17 [0–101]^#^	0.759	0.227 n.s^#^
F LVOT, AU	65 [5–238]^#^		10 [0–95]^#^		26 [0–166]^#^		0.544 n.s^#^
M LVOT density, AU/cm^2^	9 [0–48]^#^	0.235	6 [0–27]^#^	0.629	3 [0–20]^#^	0.589	0.168 n.s^#^
F LVOT density, AU/cm^2^	16 [1–56]^#^		3 [0–19]^#^		5 [0–35]^#^		0.582 n.s^#^

Values are mean ± SD, median ± interquartile range or *n* (%)

*AVA* aortic valve area, *AVC* aortic valve calcification, *AU* Agatston units, *dPmean* mean transvalvular gradient, *LCC* left coronary cusp, *LVEF* left-ventricular ejection fraction, *LVOT* left-ventricular outflow tract, *NCC* non-coronary cusp, *RCC* right coronary cusp

* *p* < 0.05

^#^Detailed differences between groups as defined

**Fig. 3:**
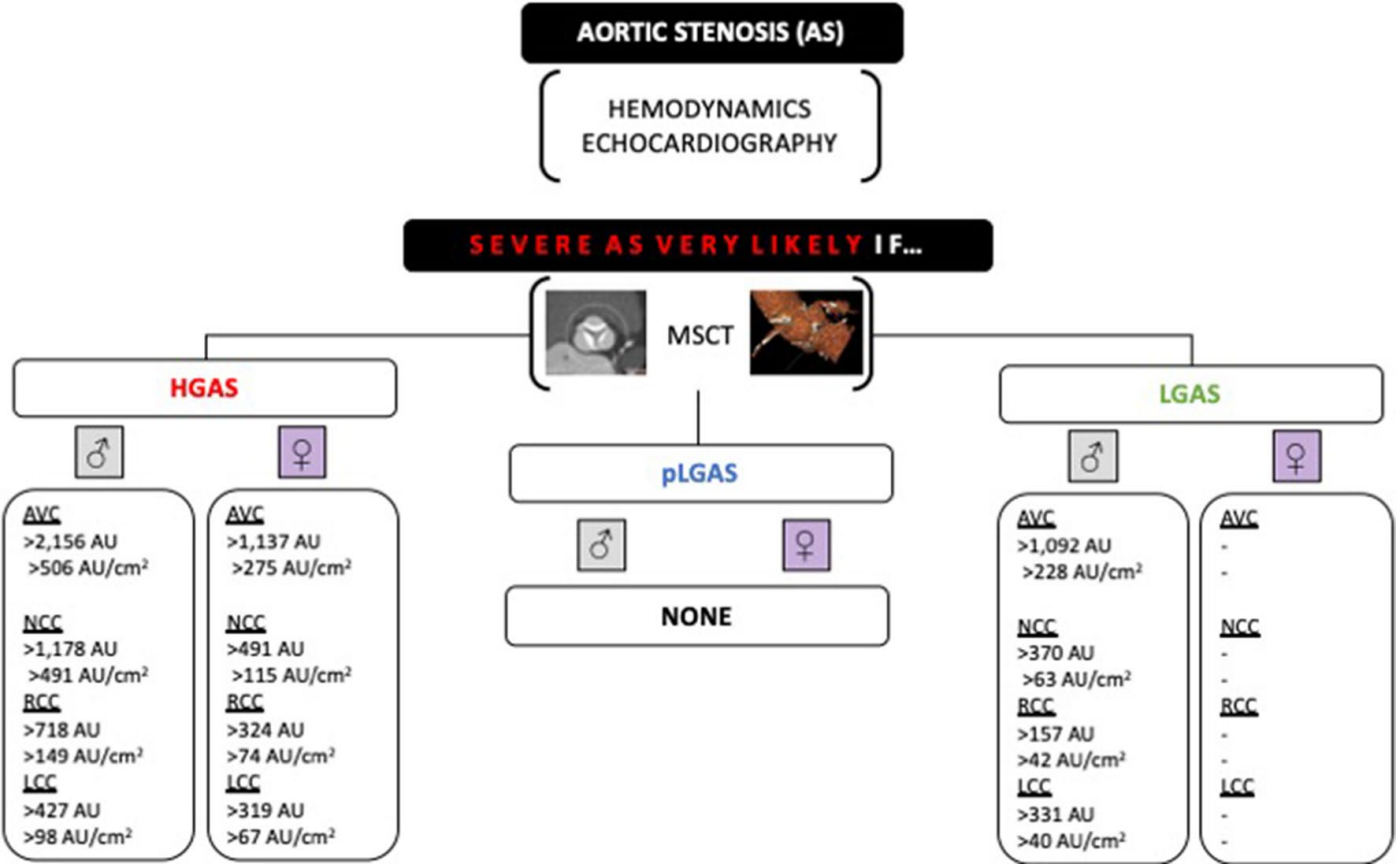
Graphical abstract—using Aortic valve calcium scoring for an individualized diagnostic severity tool in different AS entities. Primary assessment of severity and hemodynamics by echocardiography. Further discrimination into several AS entities according to current recommendations. MSCT-derived AVC can provide complementary assessment of disease severity in several AS entities

C-statistics was most reliable in male and female patients presenting with HGAS (Table 3A): an AVC > 2156 AU in men (c-index 0.76, 95% CI 0.65–0.79; *p* < 0.0001*; sensitivity 65%, specificity 79%) and > 1292 AU in women (c-index 0.85, 95% CI 0.79–0.91; *p* < 0.0001*; sensitivity 62%, specificity 93%) or AVC density > 406 AU/cm^2^ in men (c-index 0.82, 95% CI 0.76–0.89; *p* < 0.0001*; sensitivity 75%, specificity 75%) and > 259 AU/cm^2^ in women (c-index 0.86, 95% CI 0.81–0.92; *p* < 0.0001*; sensitivity 80%, specificity 80%) was found to be the best threshold for diagnosis of severe AS if compared to overall moderate AS. In adaption to LVF (</> 50%), detailed analysis of the calcium distribution in several flow patterns and moderate-to-severe AS showed that only AVC and leaflet calcification in men was able to differentiate between severe LGAS and moderate AS but not between severe pLGAS and moderate AS. For detailed information, please see Table 3B and the graphical abstract illustrating recommended aortic valve calcium scoring for an individualized diagnostic severity tool in different AS entities (Fig. 3).

**Table 3:** Discrimination performance (ROC and AUC statistics) of severe AS entities in contrast to moderate AS

Parameters	Entities	AUC	*p* value	Lower 95% CI	Upper 95% CI	Threshold	Sensitivity (%)	Specificity (%)	LR
Overall data									
AVC (AU) male	HGAS	0.76	**< 0.0001**	0.69	0.83	> 2156	65	79	3.1
	pLGAS	0.62	**0.0081**	0.53	0.71	> 1581	63	62	1.7
	LGAS	0.61	**0.0348**	0.51	0.70	> 1589	53	62	1.4
AVC (AU) female	HGAS	0.85	**< 0.0001**	0.79	0.91	> 1540	62	93	9.0
	pLGAS	0.66	**0.0012**	0.57	0.74	> 1016	50	73	1.8
	LGAS	0.62	**0.0425**	0.51	0.74	> 1012	50	73	1.8
AVC (AU/cm^2^) male	HGAS	0.82	**< 0.0001**	0.76	0.89	> 406	75	75	3.0
	pLGAS	0.63	**0.0050**	0.54	0.72	> 323	52	72	1.8
	LGAS	0.62	**0.0196**	0.54	0.72	> 322	51	72	1.8
AVC (AU/cm^2^) female	HGAS	0.86	**< 0.0001**	0.81	0.92	> 259	80	80	3.9
	pLGAS	0.66	**0.0008**	0.58	0.75	> 192	62	68	2.0
	LGAS	0.63	**0.00310**	0.52	0.74	> 180	63	66	1.8
B) Adapted to LVF									
AVC (AU) male	HGAS	0.76	**< 0.0001**	0.68	0.85	> 2156	76	72	2.8
	pLGAS	0.54	0.4108	0.44	0.65	> 1754	55	56	1.2
	LGAS	0.79	**0.0002**	0.67	0.90	> 1092	78	76	3.3
AVC (AU) female	HGAS	0.86	**< 0.0001**	0.80	0.93	> 1137	80	80	4.0
	pLGAS	0.66	**0.0022**	0.57	0.75	> 848	58	63	1.6
	LGAS	0.60	**0.3580**	0.40	0.79	> 1012	50	67	1.5
AVC (AU/cm^2^) male	HGAS	0.78	**< 0.0001**	0.70	0.86	> 506	59	78	2.7
	pLGAS	0.54	0.4314	0.44	0.65	> 320	53	61	1.4
	LGAS	0.81	**< 0.0001**	0.71	0.92	> 228	75	88	6.4
AVC (AU/cm^2^) female	HGAS	0.87	**< 0.0001**	0.81	0.93	> 275	80	83	4.6
	pLGAS	0.67	**0.0045**	0.58	0.75	> 178	65	69	2.1
	LGAS	0.63	0.2287	0.43	0.82	> 231	50	78	2.3
NCC (AU) male	HGAS	0.70	**0.0003**	0.60	0.80	> 1178	50	78	2.2
	pLGAS	0.53	0.6028	0.42	0.63	> 656	55	56	1.2
	LGAS	0.77	**0.0004**	0.65	0.89	> 370	80	76	3.4
NCC (AU) female	HGAS	0.81	**< 0.0001**	0.73	0.90	> 491	72	80	3.6
	pLGAS	0.64	**0.0074**	0.54	0.74	> 277	63	63	1.7
	LGAS	0.67	0.0985	0.48	0.87	> 286	65	78	2.9
NCC (AU/cm^2^) male	HGAS	0.70	**0.0002**	0.61	0.80	> 193	56	78	2.5
	pLGAS	0.52	0.6549	0.42	0.63	> 114	60	50	1.2
	LGAS	0.77	**0.0004**	0.65	0.90	> 63	79	76	3.3
NCC (AU/cm^2^) female	HGAS	0.83	**< 0.0001**	0.75	0.90	> 115	74	83	4.3
	pLGAS	0.64	**0.0075**	0.42	0.63	> 74	60	69	1.9
	LGAS	0.69	0.0660	0.49	0.90	> 70	63	78	2.8
RCC (AU) male	HGAS	0.76	**< 0.0001**	0.67	0.85	> 718	66	78	2.9
	pLGAS	0.55	0.6228	0.45	0.66	> 415	60	58	1.5
	LGAS	0.72	**0.0054**	0.58	0.85	> 157	89	50	1.8
RCC (AU) female	HGAS	0.82	**< 0.0001**	0.75	0.89	> 324	74	80	3.7
	pLGAS	0.68	**0.0010**	0.58	0.77	> 192	71	60	1.8
	LGAS	0.51	0.9042	0.28	0.75	< 249	54	67	1.6
RCC (AU/cm^2^) male	HGAS	0.77	**< 0.0001**	0.69	0.86	> 149	60	75	2.4
	pLGAS	0.54	0.4473	0.43	0.65	> 76	63	61	1.6
	LGAS	0.72	**0.0054**	0.58	0.85	> 42	81	59	2.0
	pLGAS	0.67	**0.0018**	0.57	0.76	> 52	63	63	1.7
	LGAS	0.53	0.7677	0.65	0.90	< 60	56	67	1.7
LCC (AU) male	HGAS	0.75	**< 0.0001**	0.66	0.85	> 427	81	67	2.4
	pLGAS	0.57	0.2068	0.46	0.67	> 423	53	67	1.6
	LGAS	0.73	**0.0032**	0.60	0.85	> 331	63	71	2.2
LCC (AU) female	HGAS	0.78	**< 0.0001**	0.70	0.86	> 319	64	83	3.8
	pLGAS	0.60	0.0621	0.50	0.70	> 230	52	60	1.3
	LGAS	0.52	0.8869	0.32	0.71	> 211	50	67	1.5
LCC (AU/cm^2^) male	HGAS	0.76	**< 0.0001**	0.67	0.85	> 98	75	69	2.5
	pLGAS	0.57	0.2100	0.46	0.67	> 74	58	53	1.2
	LGAS	0.75	**0.0012**	0.63	0.86	> 40	84	53	1.8
LCC (AU/cm^2^) female	HGAS	0.78	**< 0.0001**	0.70	0.86	> 67	73	77	3.2
	pLGAS	0.60	0.0680	0.50	0.69	> 52	54	57	1.3
	LGAS	0.54	0.7262	0.35	0.72	> 46	54	67	1.6

*AVA* aortic valve area, *AVC* aortic valve calcification, *AU* Agatston units, *AUC* area under the curve, *LVOT* left-ventricular outflow tract, *NCC* non-coronary cusp, *RCC* right coronary cusp

* *p* < 0.05

## Discussion

Existing sex- and flow-related AVC studies are limited to a small number of studies, and detailed information about calcium distribution and severity in patients with altered flow patterns is still missing. To our knowledge, this is the first study addressing AVC, including a proposal for the best thresholds to distinguish between moderate and severe AS in the three AS entities,

Our retrospective study revealed several new aspects:

1. The NCC was the most calcified cusp throughout gender, AS severity, and AS entities.
2. AVC thresholds were comparable in pLGAS and LGAS and did significantly differ from patients with HGAS, who presented with the highest calcium load.
3. LVOT calcification may be higher in severe and critical stenosis, but failed to be a reliable indicator for accelerating AS.
4. Only AVC in men was able to differentiate between severe LGAS and moderate AS.

### AVC thresholds according to AS severity

It is well known that sex-specific AVC thresholds can help to identify severe AS and provide useful prognostic information. In this context, it has been shown that women, if compared to their male counterparts, tend to reach a higher degree of stenosis for the same amount of AVC burden, even after adjusting for body surface area and smaller aortic annulus [4, 5]. AVC thresholds, including aortic valve leaflets and the LVOT, have not been separately addressed thus far.

#### AVC thresholds (in total and as density proportion)

According to previous multicenter trials and current guidelines, AVC scores are higher in male than in female patients regarding the calcium burden of the aortic valve in total, and separated into distinct leaflet calcification burden, after recalculating in density proportion. AVC thresholds increased about nearly twice AU from moderate-to-severe AS. NCC density proportion performed best in female and LCC density proportion best in male patients. In all groups of severity, the NCC was the most calcified cusp. Interestingly, LVOT calcification was consistently comparable concerning sex differences and AS severity in moderate and severe AS, and failed to be a reliable indicator for accelerating AS in borderline AS.

### AVC thresholds according to AS entity

Previous studies have demonstrated that the extent of AVC correlates well with hemodynamic severity of AS [2, 3]. Furthermore, the three different AS entities are associated with discordant mean gradients apart from the AVA, based on multiple valvular and non-valvular factors independent of flow [4, 9, 10]. However, the definition of AS entities is subject to continuous modification in the setting of borderline or severe AS, which are not in line with the recommended pressure gradients and are likewise associated with different outcomes. Nowadays, pLGAS and LGAS estimate for nearly 15–30% of patients, so additional identifying parameters are strongly required, leading to an integrated approach, considering all available functional data together, in line with the clinical presentation.

#### Discussion of AVC thresholds in different AS entities

Patients with severe AS were separated into HG, pLG, and LGAS according to existing guidelines. As expected, in severe AS, AVC scores were higher in male than in female patients and highest in HGAS with a median AVC of 1785 AU in women and 3076 AU in men or an AVC density of 424 AU/cm^2^ in women and 551 AU/cm^2^ in men, and, therefore, higher than all-over severe AS independent from flow pattern. Interestingly, AVC thresholds were consistently comparable in pLGAS (w/m: 1014 AU vs. 1893 AU; 244 AU/cm^2^ vs. 333 AU/cm^2^) and LGAS (w/m: 1007 AU vs. 1644 AU; 230 AU/cm^2^ vs. 322 AU/cm^2^), nearly achieving recommended AVC thresholds for severe AS. This is a new aspect, although several studies investigated the role of dis-concordant AS severity and AVC distribution [3, 10, 11]. If compared to overall moderate AS, c-statistics in HGAS revealed an AVC of 1292 AU in women (c-index 0.85, 95% CI 0.79–0.91; *p* < 0.0001*; sensitivity 62%, specificity 93%) and AVC of 2156 AU in men (c-index 0.76, 95% CI 0.65–0.79; *p* < 0.0001*; sensitivity 65%, specificity 79%) or AVC density of 259 AU/cm^2^ in women (c-index 0.86, 95% CI 0.81–0.92; *p* < 0.0001*; sensitivity 80%, specificity 80%) and of 406 AU/cm^2^ in men (c-index 0.82, 95% CI 0.76–0.89; *p* < 0.0001*; sensitivity 75%, specificity 75%) as the optimal threshold for diagnosis of severe AS. Concerning others’ flow pattern, only AVC and leaflet calcification in men were able to differentiate between severe LGAS and moderate AS.

#### The role of pronounced NCC calcification

As mentioned before, the NCC was the most calcified cusp through all severity grades and AS entities. C-statistics depicted best coherence of NCC-calcium load with severity of AS in women (c-index 0.73–0.83; 95% CI 0.66–0.88; *p* < 0.0001*; sensitivity 70–77%, specificity 73–80%) if compared to male counterparts. In male patients, the LCC calcium load performed even better with best coherence concerning LCC density (c-index 0.82; 95% CI 0.74–0.89; *p* < 0.0001*; sensitivity 84%, specificity 70%). Further divided into several AS entities, NCC-calcium load performed best in women with HGAS and men with LGAS. In men with HGAS, RCC calcification performed even better. LCC calcification load revealed a comparable coherence in male patients with HGAS and LGAS, but only in women with HGAS. Concerning pLGAS, all AVC thresholds failed to differentiate between severe and moderate to borderline AS. Taken all these considerations into account, the best performance of NCC calcification thresholds under altered flow conditions might be a key factor in cases with predominating fibrosis, asymmetrical leaflet calcification, and borderline AVC in total. Pronounced NCC calcium load may be one of the first markers of underestimated AS in borderline conditions. To our knowledge, this is the first study, providing detailed information on leaflet calcification load in the context of AS severity and AS entities.

#### The role of LVOT calcification

Until now, the role of LVOT calcification has only been considered in the context of aortic regurgitation, conduction disturbances, and risk for other major adverse events in patients undergoing surgical and transcatheter aortic valve replacement [12, 13]. According to practical experience, the amount of LVOT calcium load is often enhanced in severe and critical AS with an AVA ≤ 0.6 cm^2^. In our study, LVOT calcification was higher in in patients with HGAS but comparable between men and women throughout different AS entities. However, LVOT calcification may be higher in severe AS, but failed to be a reliable indicator for accelerating AS in this study.

## Conclusion

Data from this retrospective analysis indicate that the NCC shows predominating degeneration throughout gender, AS severity, and several AS entities. AVC was comparable in severe pLG and LGAS, but only AVC in severe LGAS could sufficiently distinguish moderate from severe AS in men. LVOT calcification failed to be a reliable indicator of accelerating AS.

## Limitations

This study is a single-center, retrospective analysis which is underpowered in regards of low-flow entities and, therefore, a meaningful interpretation of reliable AVC thresholds in these sub-cohorts may not be possible. AS hemodynamics is subject to several influencing factors such as blood pressure, volume status, and the underlying heart rhythm (atrial fibrillation vs sinus rhythm vs paced rhythm). A confounding factor is the small anatomy in women, which may be addressed by exclusion of increased LVOT flow and energy loss index in a small anatomy.

## Electronic supplementary material

Below is the link to the electronic supplementary material.

Supplementary file1 (DOCX 26 kb)

## Abbreviations

AS: Aortic stenosis
AVA: Aortic valve area
AU: Agatston units
LCC: Left coronary cusp
(p) LG: (Paradoxical) low gradient
LVOT: Left-ventricular outflow tract
NCC: Non-coronary cusp
HG: High-gradient
MSCT: Multislice computed tomography
RCC: Right coronary cusp
TTE: Transthoracic echocardiography
