# Supplementary material for: Aortic valve calcification is subject to aortic stenosis severity and the underlying flow pattern
Source: Heart Vessels. 2020 Sep 7;36(2):242–51. doi: 10.1007/s00380-020-01688-9 (PMC7843559; doi:10.1007/s00380-020-01688-9)
Supplement: Supplementary file 1 — Supplementary file1 (DOCX 26 kb) [file 380_2020_1688_MOESM1_ESM.docx]

1. **DATA SUPPLEMENTS**

**Table 1. Patients clinical and functional characteristics**

| **Clinical data** | **Over-all**  **(n=938)** | **Male**  **(n=443)** | **female**  **(n=495)** | **p-value** |
| --- | --- | --- | --- | --- |
| Age, years | 81.4 ± 5.7 | 80.4 ± 5.8 | 82.3 ± 5.4 | **<0.0001* |
| BMI | 26.8 ± 4.9 | 27.0 ± 4.3 | 26.6 ± 5.3 | 0.310 |
| CAD | 678 (72.3) | 357 (80.6) | 321 (64.9) | **<0.0001* |
| Previous PCI | 363 (38.7) | 208 (47.0) | 155 (31.3) | **<0.0001* |
| Previous CABG | 120 (12.8) | 98 (22.1) | 22 (4.5) | **<0.0001* |
| Previous valve | 5 (0.5) | 2 (0.5) | 3 (0.6) | 1.000 |
| Arterial hypertension | 867 (92.4) | 406 (91.6) | 461 (93.1) | 0.458 |
| PHT | 617 (65.8) | 287 (64.9) | 330 (66.7) | 0.535 |
| Diabetes mellitus | 298 (31.8) | 155 (35.0) | 143 (28.9) | 0.168 |
| PAD | 271 (28.9) | 157 (35.4) | 114 (23.0) | **<0.0001* |
| CVD | 159 (17.0) | 89 (20.1) | 70 (14.1) | **0.018* |
| Previous RRT | 40 (4.3) | 26 (5.9) | 14 (2.8) | **0.024* |
| COPD | 292 (31.1) | 143 (32.3) | 149 (30.1) | 0.418 |
| Atrial fibrillation | 350 (37.3) | 166 (37.5) | 184 (37.2) | 0.946 |
| **Functional data** |  |  |  |  |
| Log ES_I, % | 25.7 ± 18.8 | 25.7 ± 16.6 | 25.7 ± 20.6 | 0.969 |
| LVEF, % | 55.1 ± 13.0 | 52.0 ± 13.4 | 58.2 ± 12.7 | **<0.0001* |
| CI, l/min/m^2^ | 2.4 ± 1.5 | 2.3 ± 1.3 | 2.4 ± 1.7 | 0.610 |
| PAsys, mmHg | 44.7 ± 16.5 | 44.4 ± 16.3 | 45.1 ± 16.7 | 0.581 |
| LVEDP, mmHg | 22.7 ± 10.1 | 22.6 ± 11.3 | 22.8 ± 9.0 | 0.825 |
| AVA, cm^2^ | 0.8 ± 0.2 | 0.8 ± 0.2 | 0.7 ± 0.2 | **<0.0001* |
| dPmax, mmHg | 60.3 ± 23.7 | 57.8 ± 21.2 | 62.4 ± 25.6 | **0.003* |
| dPmean, mmHg | 37.2 ± 16.0 | 35.7 ± 14.9 | 38.6 ± 16.7 | **0.005* |
| **MSCT data** |  |  |  |  |
| Annulus area, cm^2^ | 5.0 ± 2.4 | 5.6 ± 2.5 | 4.5 ± 2.1 | **<0.0001* |
| AVC, AU | 1,627 [917-2,716] | 2,155 [1,339-3,230] | 1,309 [698-2,094] | **<0.0001* |
| AVC density, AU/cm^2^ | 340 [199-539] | 388 [243-577] | 307 [168-474] | **<0.0001* |
| NCC, AU | 659 [327-1,208] | 820 [473-1342] | 535 [240-974] | **<0.0001* |
| NCC density, AU/cm^2^ | 134 [70-234] | 150 [90-241] | 121 [57-229] | **0.0001* |
| RCC, AU | 456 [232-841] | 617 [329-1,048] | 344 [171-629] | **<0.0001* |
| RCC density, AU/cm^2^ | 94 [50-165] | 108 [61-181] | 77 [41-147] | **<0.0001* |
| LCC, AU | 411 [215-777] | 580 [303-968] | 308 [159-603] | **<0.0001* |
| LCC density, AU/cm^2^ | 86 [46-154] | 104 [55-169] | 76 [37-141] | **<0.0001* |
| LVOT, AU | 26 [0-176] | 30 [0-168] | 22 [0-181] | 0.925 |
| LVOT density, AU/cm^2^ | 5 [0-34] | 6 [0-29] | 5 [0-38] | 0.413 |
| Values are mean ± SD, median ± interquartile range or n (%).  AF=atrial fibrillation; AU=Agatston units; AVA(i)=aortic valve area (indexed); BMI=body mass index; CABG=coronary artery bypass graft; CAD=coronary artery disease; CI=cardiac index; COPD=chronic obstructive pulmonary disease; CVD=cerebrovascular disease; dPmean/max=mean/max. transvalvular gradient; LCC=Left coronary cusp; LVEDP=Left ventricular enddiastolic pressure; LVEF=Left ventricular ejection fraction; LVOT=Left ventricular outflow tract; NCC=Non-coronary cusp; PCI=percutaneous coronary intervention; PHT=pulmonary hypertension; PAD=peripheral artery disease; RCC=Right coronary cusp; RRT=renal replacement therapy; | | | | |

**Table 2. Discrimination performance (ROC and AUC statistics) AS-severity (moderate-to-severe)**

| **Parameters** | **Entities** | **AUC** | **p-value** | **Lower 95%-CI** | **Upper 95%-CI** | **Threshold** | **Sensitivity (%)** | **Specificity (%)** | **LR** |
| --- | --- | --- | --- | --- | --- | --- | --- | --- | --- |
| **AVC (AU)** | **male** | 0.70 | **<0.0001** | 0.62 | 0.77 | >2,020 | 58 | 74 | 2.2 |
|  | **female** | 0.75 | **<0.0001** | 0.68 | 0.81 | >1,137 | 62 | 77 | 2.7 |
|  |  |  |  |  |  |  |  |  |  |
| **AVC (AU/cm^2^)** | **male** | 0.70 | **<0.0001** | 0.63 | 0.78 | >323 | 64 | 72 | 2.2 |
|  | **female** | 0.75 | **<0.0001** | 0.69 | 0.82 | >202 | 72 | 68 | 2.3 |
|  |  |  |  |  |  |  |  |  |  |
| **NCC (AU)** | **male** | 0.66 | **<0.0001** | 0.59 | 0.74 | >624 | 66 | 64 | 1.8 |
|  | **female** | 0.83 | **<0.0001** | 0.79 | 0.88 | >252 | 77 | 80 | 3.8 |
|  |  |  |  |  |  |  |  |  |  |
| **NCC (AU/cm^2^)** | **male** | 0.66 | **0.0001** | 0.59 | 0.74 | >115 | 66 | 62 | 1.7 |
|  | **female** | 0.73 | **<0.0001** | 0.66 | 0.81 | >76 | 70 | 73 | 2.6 |
|  |  |  |  |  |  |  |  |  |  |
| **RCC (AU)** | **male** | 0.68 | **<0.0001** | 0.60 | 0.75 | >619 | 53 | 73 | 2.0 |
|  | **female** | 0.71 | **<0.0001** | 0.63 | 0.79 | >306 | 57 | 75 | 2.3 |
|  |  |  |  |  |  |  |  |  |  |
| **RCC (AU/cm^2^)** | **male** | 0.69 | **<0.0001** | 0.61 | 0.76 | >89 | 63 | 68 | 2.0 |
|  | **female** | 0.71 | **<0.0001** | 0.63 | 0.78 | >67 | 62 | 66 | 1.8 |
|  |  |  |  |  |  |  |  |  |  |
| **LCC (AU)** | **male** | 0.68 | **<0.0001** | 0.61 | 0.76 | >504 | 60 | 72 | 2.1 |
|  | **female** | 0.67 | **0.0001** | 0.60 | 0.75 | >315 | 52 | 80 | 2.5 |
|  |  |  |  |  |  |  |  |  |  |
| **LCC (AU/cm^2^)** | **male** | 0.82 | **<0.0001** | 0.74 | 0.89 | >210 | 84 | 70 | 2.7 |
|  | **female** | 0.68 | **0.0001** | 0.60 | 0.75 | >78 | 51 | 80 | 2.5 |
|  |  |  |  |  |  |  |  |  |  |
| **LVOT (AU)** | **male** | 0.52 | 0.6681 | 0.44 | 0.60 | >277 | 19 | 85 | 1.3 |
|  | **female** | 0.60 | **0.0306** | 0.51 | 0.68 | >239 | 22 | 91 | 2.4 |
|  |  |  |  |  |  |  |  |  |  |
| **LVOT (AU/cm^2^)** | **male** | 0.52 | 0.6306 | 0.44 | 0.60 | >32 | 25 | 79 | 1.2 |
|  | **female** | 0.60 | **0.0266** | 0.52 | 0.69 | >38 | 27 | 86 | 2.0 |
